# FOXP2+ Chief Cells and CXCL14+ Fibroblasts Drive Fibrotic Remodeling in Carotid Body Tumors

**DOI:** 10.3390/ijms27135750

**Published:** 2026-06-25

**Authors:** Kangxi Cao, Jiazhi Yu, Guangnan Ao, Zongli Han, Zhongzheng Wang, Yunfeng Han, Tao Wang

**Affiliations:** Department of Neurosurgery, Peking University Third Hospital, Beijing 100191, China

**Keywords:** carotid body tumor, fibrosis, single-cell RNA sequencing, FOXP2, CXCL14, tumor microenvironment

## Abstract

Carotid body tumors (CBTs) exhibit pronounced clinical heterogeneity, particularly in fibrotic progression, yet the underlying cellular mechanisms remain poorly defined. Here, we performed single-cell RNA sequencing on 64,944 cells from three fibrotic CBT (FCBT) and three non-fibrotic CBT (nFCBT) specimens to construct a high-resolution cellular atlas of CBT fibrosis. Integrated analyses revealed that FCBTs are distinguished by a FOXP2+ chief cell subpopulation exhibiting a metabolic shift toward mitochondrial respiration and enhanced MIF signaling, which may facilitate macrophage recruitment. Endothelial cells expanded in FCBTs and acquired pro-angiogenic signatures driven by macrophage-derived CXCL signaling. Notably, CXCL14+ fibroblasts emerged as the principal effectors of extracellular matrix deposition, with lineage inference suggesting their origin from smooth muscle cells. Immune cells, including T/NK and mast cells, further modulated the fibrotic niche through cytokine interactions. This study provides the first comprehensive single-cell dissection of CBT fibrosis, identifies FOXP2+ chief cells as initiators of stromal remodeling, and highlights CXCL14+ fibroblasts as key matrix-producing effectors. These findings nominate FOXP2 and CXCL14 as potential therapeutic targets for mitigating fibrosis in CBT patients.

## 1. Introduction

Carotid body tumors (CBTs), also known as chemodectoma, account for approximately 60–70% of all head and neck paragangliomas [1,2]. Research over the past decades has primarily focused on hypoxia [1,3], SDH family mutations [4], clinical management [5], and surgery-related complications [6]. However, the significant heterogeneity inherent to CBTs has received less attention. Our previous study exploring neuronal injury post-CBT resection found that patients with firm, fibrotic tumors were more susceptible to surgery-related neurological complications [6].

The Shamblin classification system [7] is widely used to describe CBTs [8,9], with higher grades generally indicating greater surgical difficulty. Based on over 500 CBT resections, we observed that Shamblin grade does not consistently predict intraoperative fibrosis. Some Shamblin I CBTs are highly fibrotic and severely invasive to the carotid artery, while some Shamblin III CBTs are minimally fibrotic and easily dissected from the arterial surface. This marked inter-sample heterogeneity raises critical questions: Why do CBTs exhibit such variability? What molecular events occur during their development?

Fibrosis represents the body’s reparative response to injury [10]. Chronic injury from diverse etiologies leads to persistent fibrosis [11] and progressive ECM accumulation [12] within tissues. During fibrosis, stromal cells release pro-angiogenic factors such as matrix metalloproteinases (MMPs), transforming growth factor-β (TGF-β), and vascular endothelial growth factor (VEGF) [13,14]. These factors promote epithelial cell division and proliferation [15], enhance vascular permeability [16], disrupt basement membrane-ECM connections [17], facilitate pathological vessel formation [18], and thereby contribute to fibrosis.

Current understanding of CBT pathophysiology remains obscure, primarily relying on histomorphology, bulk gene analysis, and immunohistochemistry, partly due to the lack of representative laboratory models [19,20,21]. Single-cell RNA sequencing (scRNA-seq) has emerged as a powerful tool for defining cellular composition (including novel or rare subpopulations), cell-specific transcriptome dynamics, cell lineage tracing, and cell-cell communication within complex tissues at high resolution [22,23,24,25]. To our knowledge, no multi-sample scRNA-seq study investigating CBT pathogenesis has been reported. Here, we present an scRNA-seq analysis of 64,944 cells from 3 fibrotic CBT (FCBT) lesions and 3 non-fibrotic CBT (nFCBT) lesions. This single-cell resolution analysis provides a comprehensive atlas of the cellular complexity and diversity within CBTs.

## 2. Results

### 2.1. scRNA-Seq Atlas of Cell Populations in CBT

**Figure 1:**
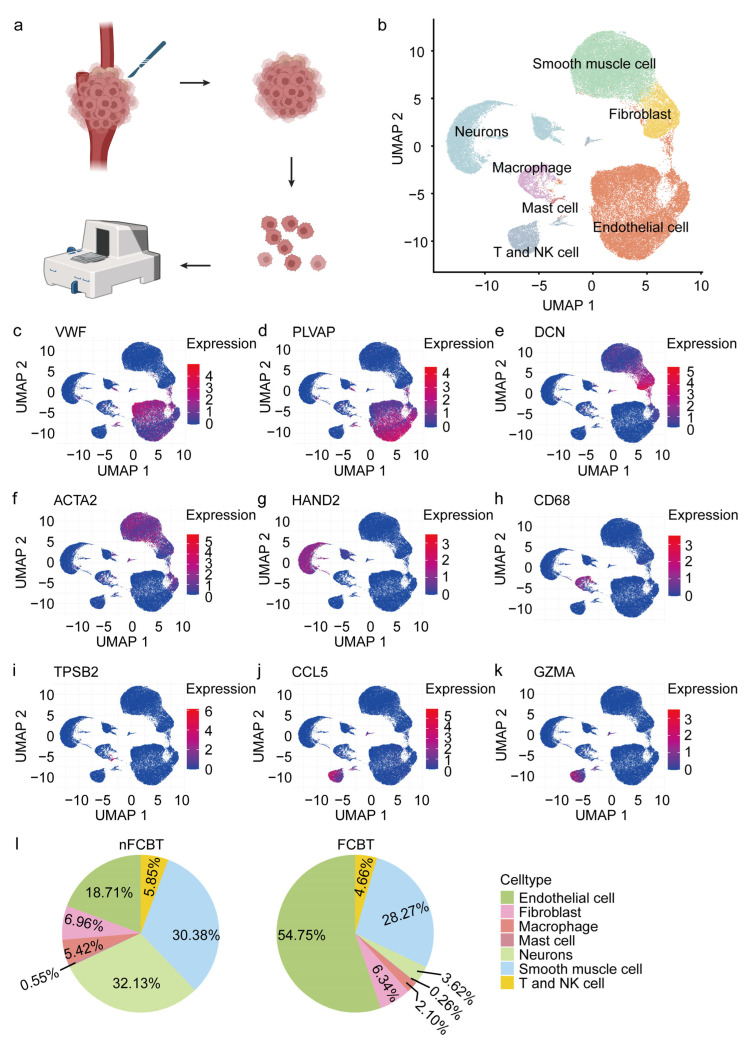
The single-cell atlas of CBT. (**a**) Schematic of single-cell isolation and RNA-seq workflow. (**b**) UMAP plot showing 7 major cell types in CBTs (n = 6). (**c**–**k**) Feature plots showing expression of marker genes: endothelial cell (VWF, PLVAP), fibroblast (DCN), smooth muscle cell (ACTA2), neurons (HAND2), macrophage (CD68), mast cell (TPSB2), and T and NK cell (CCL5, GZMA). (**l**) Pie charts showing cell type proportions in nFCBT and FCBT.

We obtained surgically resected fibrotic and non-fibrotic CBT specimens from 6 patients (3 fibrotic, 3 non-fibrotic). Although our sample size (n = 3 per group) is modest, the identified cell subpopulations and inter-group differences were consistently observed across all biological replicates, supporting the robustness of the main conclusions (Supplementary Figure S1b). ScRNA-seq was performed on these specimens to dissect cellular and molecular changes (Figure 1a). After quality control (Methods), 64,944 cells were retained for analysis. Integration using the Seurat R package identified 24 clusters (c0–c23), distinguished by unique marker genes (Supplementary Figure S1a). Based on canonical marker expression (Figure 1c–k), the 24 clusters were annotated as 7 major cell types (Figure 1b): endothelial cells (ECs) (VWF+), fibroblasts (FIBs) (DCN+), macrophages (MACs) (CD68+), neurons (NEUs) (HAND2+), smooth muscle cells (SMCs) (ACTA2+), T and NK cells (CCL5+, GZMA+), and mast cells (TPSB2+). Quantification revealed significant alterations in cellular composition between FCBT and nFCBT (Figure 1l). CBT stromal cells comprised neurons, SMCs, fibroblasts, and ECs (Figure 1b,l). Immune cell infiltrates consisted of T/NK cells, macrophages, and mast cells (Figure 1b,l). EC proportions were significantly increased in FCBT, while neuron and macrophage proportions were significantly decreased. These results delineate the cellular composition and alterations in CBTs. Notably, although one nFCBT sample (nFCBT3) showed a relatively higher proportion of neuronal cells, all key findings (e.g., FOXP2+ cluster enrichment, CXCL14+ fibroblast expansion) remained consistent when pairwise sample-wise comparisons were performed, indicating that the observed differences are robust to sample-to-sample variation.

### 2.2. FOXP2+ Carotid Body Chief Cells Drive Fibrotic Microenvironment in CBT via MIF-Mediated Macrophage Recruitment

**Figure 2:**
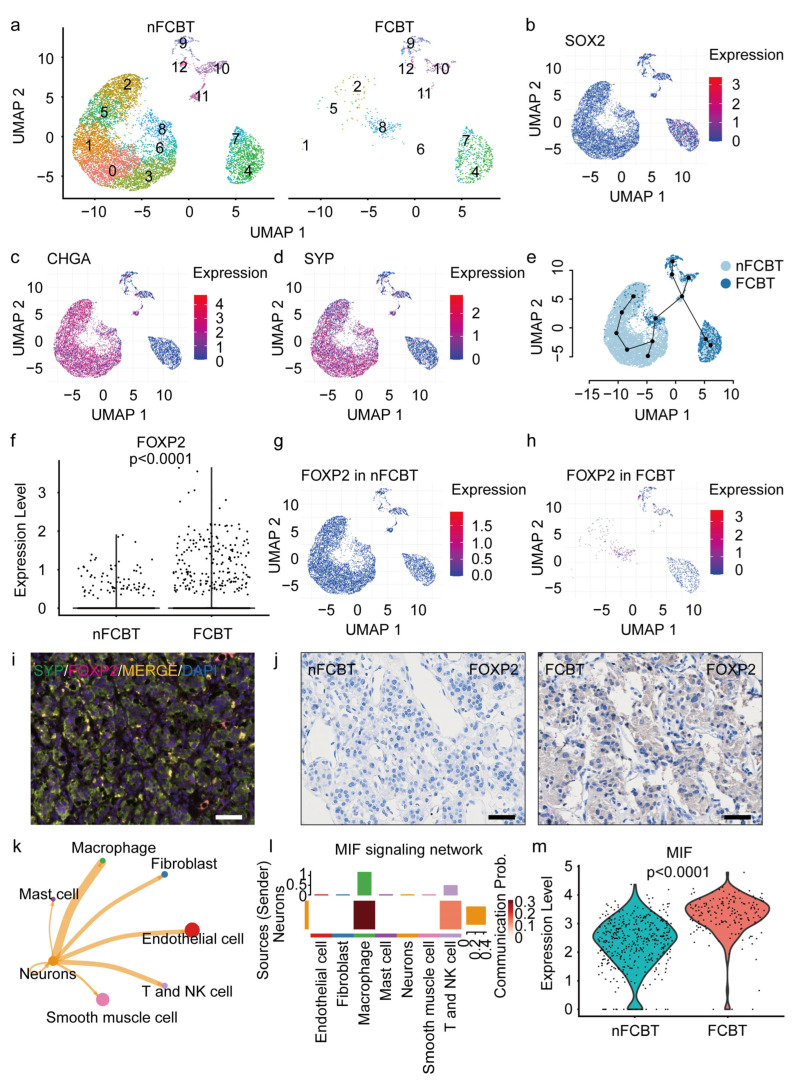
FOXP2+ chief cell marks FCBT. (**a**) UMAP plot showing neuron subclusters in nFCBT and FCBT. (**b**) Feature plot showing sustentacular cell marker SOX2. (**c**,**d**) Feature plots showing chief cell markers CHGA, SYP. (**e**) Pseudotime trajectory of neuron subclusters. (**f**) Violin plot showing FOXP2 expression in nFCBT vs. FCBT neurons, *p* < 0.0001. (**g**,**h**) Feature plots showing FOXP2 expression in nFCBT and FCBT neurons. (**i**) Multiplex IF for FOXP2 (red) and SYP (green) in CBTs (n = 10), scale bar = 50 μm. (**j**) IHC for FOXP2 on nFCBT and FCBT sections (n = 10 per group), scale bar = 50 μm. (**k**) Cell interaction network: neurons and other cells. (**l**) Cell interaction strength for MIF signaling between neurons and other cells. (**m**) Violin plot showing MIF expression in nFCBT vs. FCBT neurons, *p* < 0.0001. Data: mean ± SEM; *p*-value determined by two-tailed unpaired *t*-test (nFCBT vs. FCBT) (**f**,**m**).

Neurons are the predominant cell type in CBTs, but their role in pathogenesis is unclear. Subclustering analysis segregated neurons into 13 subclusters (Figure 2a). Hierarchical clustering divided neurons into two subpopulations based on specific markers: chief cells (SYP+, CHGA+) and sustentacular cells (SOX2+). Clusters 4 and 7 were sustentacular cells; the others were chief cells (Figure 2b–d). Chief cell composition differed significantly between FCBT and nFCBT: clusters 0, 1, 2, 3, 5, and 6 were scarce in FCBT compared to nFCBT. Pseudotime analysis identified cluster 8 as the starting point (Figure 2e). In FCBT, chief cells were predominantly cluster 8 cells, which showed minimal differentiation into other chief cell types.

To investigate this difference, Gene Ontology (GO) analysis of neuron cluster 8 revealed distinct functions: in nFCBT, the top biological processes involved hypoxia and oxygen level responses, whereas in FCBT, they related to mitochondrial composition and aerobic respiration (Supplementary Figure S2a,b), indicating a functional shift. Differential expression analysis identified Forkhead box p2 (FOXP2) as widely expressed in FCBT cluster 8 but rarely in nFCBT cluster 8 (Figure 2f–h). Immunofluorescence (IF) staining for SYP and FOXP2 (Figure 2i) and immunohistochemistry (IHC) for FOXP2 (Figure 2j and Supplementary Figure S7c) in CBT specimens confirmed these findings, with FOXP2 strongly expressed in FCBT. Further analysis of FOXP2 was performed using RNA-seq data from CBT samples. The 32 CBT samples were divided into two groups based on the median expression level of FOXP2: a FOXP2-low group and a FOXP2-high group. Differential expression analysis revealed that genes associated with fibrosis, such as COL1A1, COL3A1, and the fibroblast marker DCN, were significantly up-regulated in the FOXP2-high group (Supplementary Figure S2c). GO (Supplementary Figure S2d) and Disease Ontology (DO) (Supplementary Figure S2e) analyses of the differentially expressed genes (DEGs) indicated significant enrichment in terms related to the extracellular matrix and collagen, which are influenced by FOXP2. These findings further support the important role of FOXP2 in promoting fibrosis in CBT.

CellChat analysis of neuron interactions with other cell types (Figure 2k and Supplementary Figure S5) demonstrated robust communication between neurons and macrophages, primarily mediated by the MIF-(CD74+CD44) signaling axis. This suggests a potential role for neurons in recruiting immune cells within the macrophage-enriched tumor microenvironment. The interaction probability analysis for MIF signaling (Figure 2l) confirmed neurons as the strongest communicators with macrophages. Furthermore, MIF expression was significantly higher in FOXP2+ neurons within FCBTs compared to nFCBTs (Figure 2m), providing a potential mechanistic explanation for the development of fibrosis in these tumors.

### 2.3. Endothelial Cell Subpopulations in CBT

**Figure 3:**
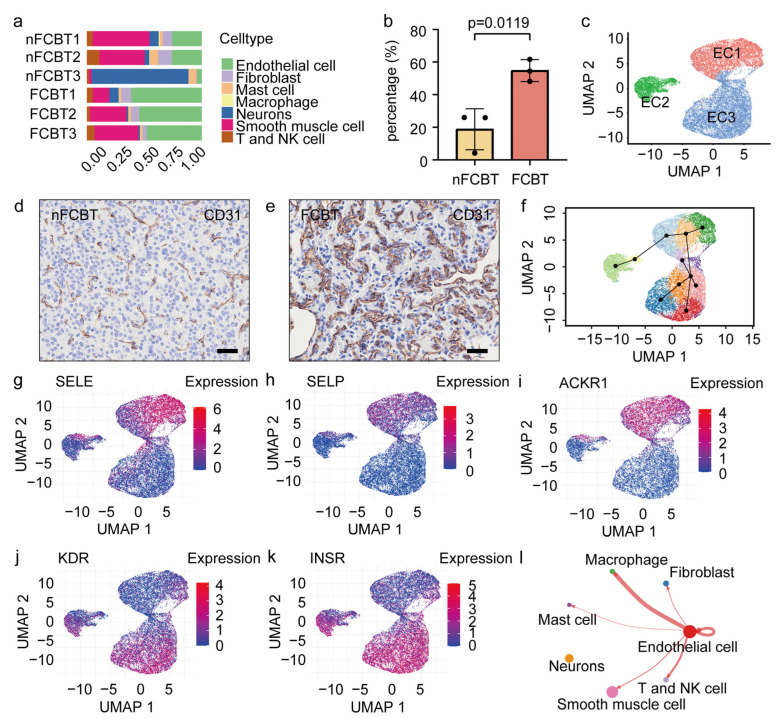
Endothelial cell heterogeneity in CBT. (**a**) Bar graph showing EC subpopulation percentages. (**b**) Percentage of total cells that are ECs (n = 3 per group, scRNA-seq data), *p* = 0.0119 (nFCBT vs. FCBT). (**c**) UMAP plot showing EC subclusters. (**d**,**e**) IHC for CD31 on nFCBT and FCBT sections (n = 10 per group), respectively; scale bar = 50 μm. (**f**) Pseudotime trajectory of EC subclusters. (**g**–**k**) Feature plots showing SELE, SELP, ACKR1, KDR, and INSR expression. (**l**) Cell interaction network: endothelial cells and other cells. Data: mean ± SEM; *p*-value determined by two-tailed unpaired *t*-test (nFCBT vs. FCBT) (**b**).

**Figure 4:**
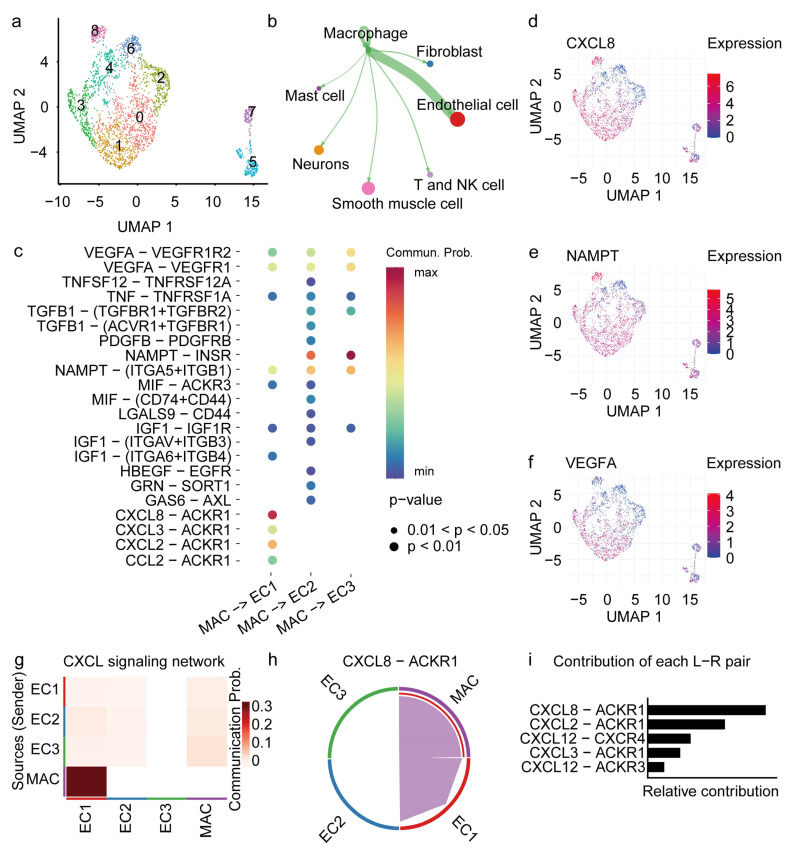
Macrophages promote angiogenesis via CXCL signaling. (**a**) UMAP plot showing macrophage subclusters. (**b**) Cell interaction network: macrophages and other cells. (**c**) Bubble plot: ligand-receptor interactions between macrophages and EC subsets. (**d**–**f**) Feature plots showing CXCL8, NAMPT, and VEGFA expression. (**g**) Cell interaction strength for CXCL signaling between macrophages and ECs. (**h**) Loop plot: CXCL8-ACKR1 interaction between macrophages and ECs. (**i**) Bar plot: contribution of individual ligand-receptor pairs to macrophage-EC communication.

As highly vascularized tumors, ECs play crucial roles in CBT formation and progression. EC proportions were significantly increased in FCBT compared to nFCBT (Figure 3a,b), and IHC staining also confirmed that there were more endothelial cells in the FCBT microenvironment (Figure 3d,e and Supplementary Figure S7d). To define EC subsets, scRNA-seq identified 11 EC subclusters (Supplementary Figure S1c). Clusters 0, 3, and 6 were grouped as EC1; clusters 2 and 10 as EC2; clusters 1, 4, 5, 7, 8, and 9 as EC3 (Figure 3c). Pseudotime analysis suggested EC2 and EC3 derive from EC1 (Figure 3f). EC1 co-expressed adhesion markers E-selectin (SELE) (Figure 3g) and P-selectin (SELP) (Figure 3h), characteristic of activated endothelium facilitating adhesion and transmigration [26]. Cell chat analysis revealed endothelial cells communicate with macrophages, fibroblasts, mast cells, smooth muscle cells, T and NK cells, and themselves (Figure 2k, Figure 3l, Figure 4b, Figure 7e and Supplementary Figure S7a,b), and ECs have a higher probability of communicating with macrophages. And endothelial cells can receive signals from all 6 other kinds of cells, mainly macrophages. EC1 also expressed the chemokine receptor ACKR1 (Figure 3i), which binds macrophage-derived CXCLs, activating inflammatory pathways and promoting EC proliferation/differentiation. EC2 and EC3 expressed markers associated with angiogenesis and endothelial regeneration, such as kinase insert domain receptor (KDR; VEGFR2) and insulin receptor (INSR) (Figure 3j,k), suggesting roles in neovascularization potentially initiated by adhesion to EC1.

### 2.4. Macrophages Promote Endothelial Cell Proliferation and Differentiation via CXCL Signaling Pathway

Macrophages were the predominant infiltrating immune cell type and were recruited to the microenvironment by neurons. Subclustering identified 9 macrophage subclusters (Figure 4a). CellChat analysis revealed strong macrophage communication with ECs (Figure 4b), particularly via specific ligand-receptor pairs (Figure 4c). Macrophages exhibited robust communication with early-stage EC1 via CXCLs-ACKR1, especially CXCL8-ACKR1; CXCL8 was widely expressed in macrophages (Figure 4d). Conversely, CXCLs-ACKR1 communication with EC2/EC3 was minimal, indicating macrophage promotion of early EC proliferation/differentiation. Macrophages also communicated with EC2/EC3 via NAMPT-INSR (absent in EC1; Figure 4e) and with all ECs via VEGFA-VEGFR (increasing probability from EC1 to EC3, likely reflecting KDR expression levels; Figure 4f). These results confirm macrophages promote EC proliferation/differentiation, with CXCL signaling being pivotal. CXCL signaling primarily mediated macrophage-to-EC1 communication, with CXCL2/3/8-ACKR1 being the dominant pair (Figure 4g–i).

### 2.5. CXCL14+ Fibroblasts Are the Main Cause of CBT Fibrosis

**Figure 5:**
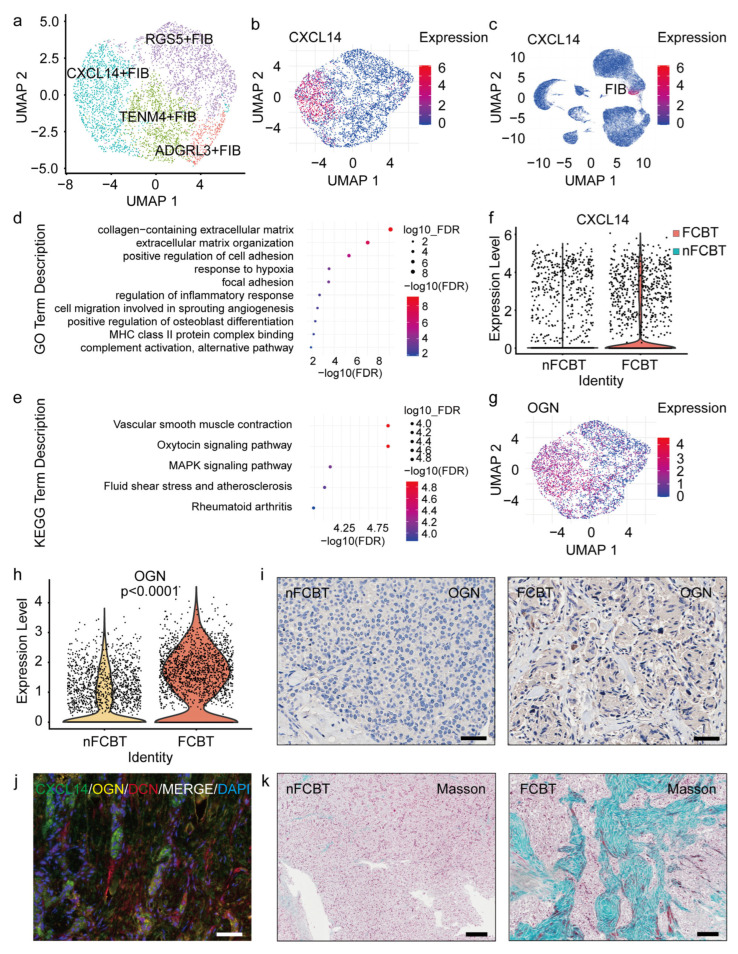
CXCL14+ fibroblasts drive CBT fibrosis. (**a**) UMAP plot showing fibroblast subclusters. (**b**,**c**) Feature plots showing CXCL14 expression in fibroblasts and all cells. (**d**) Top enriched GO pathways in CXCL14+ FIBs (GSEA). (**e**) Top enriched KEGG pathways in CXCL14+ FIBs (GSEA). (**f**) Violin plot: CXCL14 expression in nFCBT vs. FCBT fibroblasts. (**g**) Feature plot: OGN expression in fibroblasts. (**h**) Violin plot: OGN expression in nFCBT vs. FCBT fibroblasts, *p* < 0.0001. (**i**) IHC for OGN on nFCBT and FCBT sections (n = 10 per group); scale bar = 50 μm. (**j**) Multiplex IF for CXCL14 (green), OGN (yellow), and DCN (red) in CBT (n = 10); scale bar = 50 μm. (**k**) Masson staining of nFCBT and FCBT sections (n = 10 per group); collagen = blue; scale bar = 50 μm. Data: mean ± SEM; *p*-value determined by two-tailed unpaired *t*-test (nFCBT vs. FCBT) (**h**). NES: enrichment score; GSEA FDR was from Benjamini–Hochberg FDR correction (**d**,**e**).

Fibroblasts (FIBs) synthesize connective tissue ECM, maintaining structural integrity, but their role in CBT fibrosis was unknown. Subclustering identified 4 fibroblast subpopulations, and we defined them using the top differentially expressed markers: CXCL14+, TENM4+, RGS5+, and ADGRL3+ FIBs (Figure 5a and Supplementary Table S1). CXCL14+ FIBs represented a unique cluster (Figure 5b,c). GO and KEGG analyses revealed CXCL14+ FIBs were enriched for collagen biosynthesis, ECM organization, and inflammatory pathways (Figure 5d,e), defining them as inflammatory fibroblasts. CXCL14+ FIBs were significantly more abundant in FCBT than nFCBT (Figure 5f), suggesting a primary role in fibrosis. Among collagen-related genes, Osteoglycin (OGN) was upregulated in CXCL14+ FIBs (Figure 5g) and overall in FCBT fibroblasts (Figure 5h). IHC confirmed OGN upregulation in FCBT (Figure 5i and Supplementary Figure S7e). Multiplex IF confirmed CXCL14, OGN, and fibroblast marker DCN co-expression (Figure 5j). Masson staining confirmed extensive collagen deposition (green) in FCBT compared to nFCBT (Figure 5k).

### 2.6. Smooth Muscle Cells as Fibroblast Precursors

**Figure 6:**
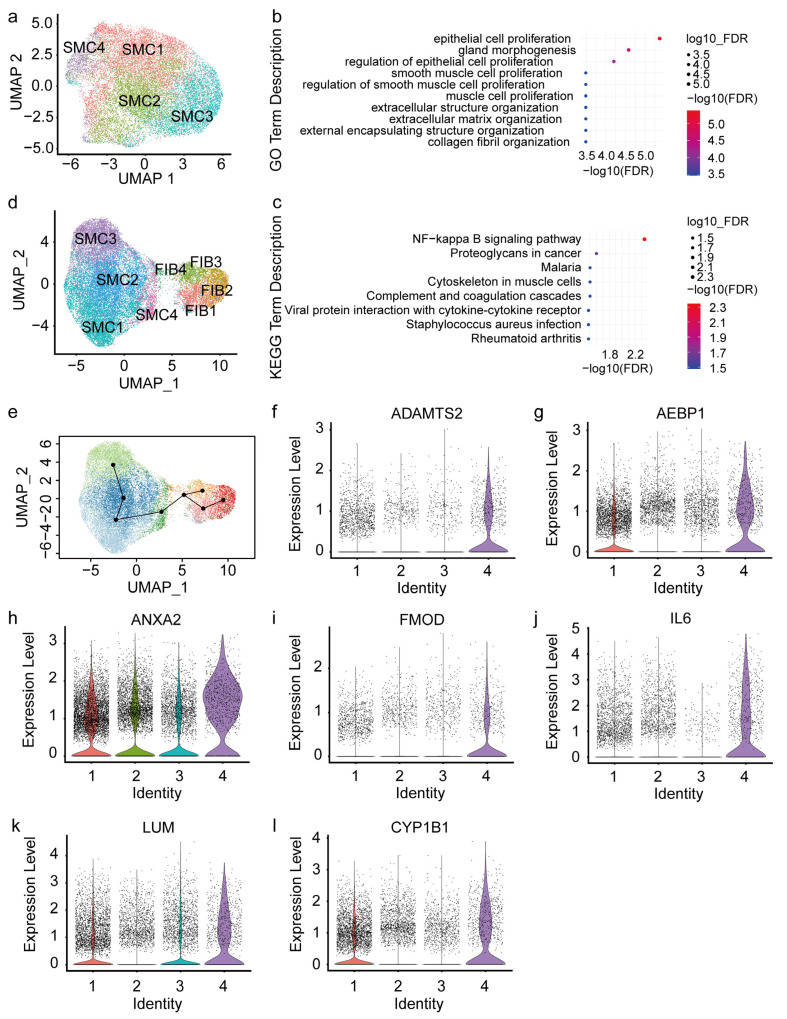
Smooth muscle cells as fibroblast precursors. (**a**) UMAP plot showing SMC subclusters. (**b**) Top enriched GO pathways in SMC4 (GSEA). (**c**) Top enriched KEGG pathways in SMC4 (GSEA). (**d**) UMAP plot: combined SMC and fibroblast subclusters. (**e**) Pseudotime trajectory: SMCs to fibroblasts. (**f**–**l**) Feature plots showing collagen fibril organization genes (ADAMTS2, AEBP1, ANXA2, FMOD, IL6, LUM, CYP1B1) expression in fibroblasts. NES: enrichment score; GSEA FDR was from Benjamini–Hochberg FDR correction (**b**,**c**).

UMAP dimensionality reduction suggested homology between SMCs and fibroblasts (Figure 1b). SMC subclustering identified 4 subclusters (SMC1–4; Figure 6a). GO/KEGG analysis revealed SMC4 was uniquely associated with collagen fibril organization and ECM pathways, similar to fibroblasts (Figure 6b,c and Supplementary Figure S3). Combined subclustering of SMCs and fibroblasts (Figure 6d) and pseudotime analysis indicated fibroblasts derive from SMC4 (Figure 6e). Expression analysis confirmed collagen fibril organization genes (ADAMTS2, AEBP1, ANXA2, FMOD, IL6, LUM, CYP1B1) were broadly and highly expressed in SMC4, supporting the SMC origin of fibroblasts (Figure 6f–l).

### 2.7. Immune Cell Populations in CBT

**Figure 7:**
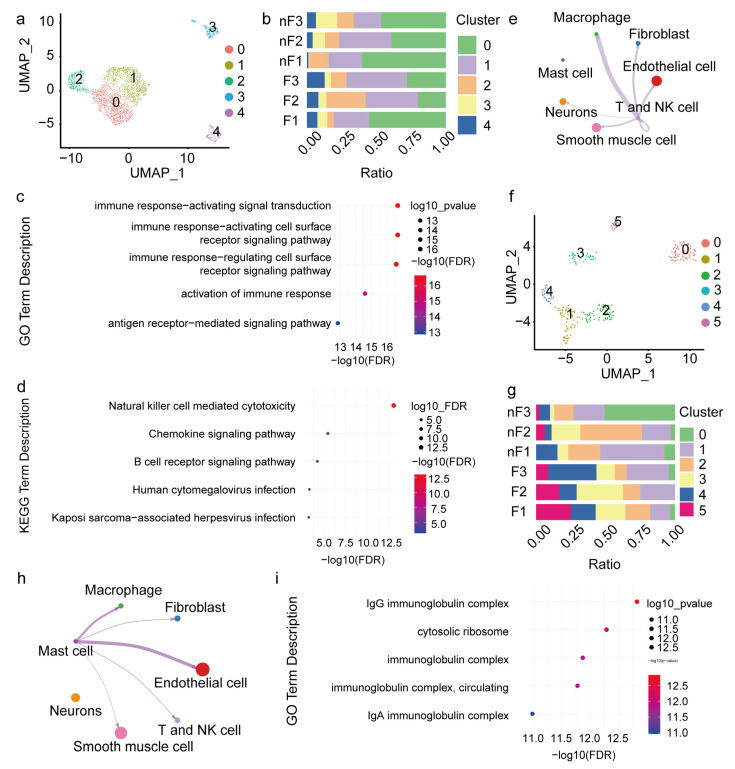
T and NK cells and mast cells promote inflammation in CBT. (**a**) UMAP plot: T and NK cell subclusters. (**b**) Bar graph: T and NK subcluster proportions. (**c**) Top enriched GO pathways in T and NK cluster 2 (GSEA). (**d**) Top enriched KEGG pathways in T and NK cluster 2 (GSEA). (**e**) Cell interaction network: T and NK cells and other cells. (**f**) UMAP plot: mast cell subclusters. (**g**) Bar graph: mast cell subcluster proportions. (**h**) Cell interaction network: mast cells and other cells. (**i**) Top enriched GO pathways in mast cell cluster 5 (GSEA). NES: enrichment score. GSEA FDR was from Benjamini–Hochberg FDR correction (**c**,**d**,**i**).

Immune dysregulation characterizes CBTs, but single-cell level changes were undefined. Besides macrophages, T and NK cells and mast cells were significant infiltrates. T and NK cells were subclustered (Figure 7a); proportions showed no significant differences between FCBT and nFCBT (Figure 7b). GO/KEGG analysis linked T and NK cluster 2 to inflammation, cluster 3 to ECM, and cluster 4 to endothelium development (Figure 7c,d and Supplementary Figure S4). CellChat analysis indicated T and NK cells communicate with ECs via CCL5-ACKR1, with fibroblasts via TGFB1-(ACVR1+TGFBR1), and with macrophages via CCL5-CCR1 (Figure 7e and Supplementary Figure S5).

Mast cells constituted only 0.55% (nFCBT) and 0.26% (FCBT) of cells. Subclustering identified 5 mast cell clusters (Figure 7f) with no significant proportional differences between groups (Figure 7g). CellChat indicated stronger mast cell communication with macrophages and ECs than with other cells (Figure 7h). GO analysis suggested mast cell cluster 5 may promote angiogenesis and fibrosis via immune processes (Figure 7i and Supplementary Figure S6).

## 3. Discussion

Carotid body tumors (CBTs) exhibit profound clinical and pathological heterogeneity, particularly in fibrotic progression, which complicates surgical intervention and patient prognosis. Our single-cell RNA sequencing (scRNA-seq) analysis of 64,944 cells from fibrotic and non-fibrotic lesions provides a detailed cellular atlas that complements and extends the recent scRNA-seq study of CBTs by Cai et al. [3], which described cell populations but did not specifically address fibrotic heterogeneity. We demonstrate that fibrotic transformation in CBTs is orchestrated by dynamic interactions between specialized cell populations: FOXP2+ chief cells initiate metabolic reprogramming and immune recruitment, endothelial cell (EC) subpopulations drive pathological angiogenesis, CXCL14+ fibroblasts execute extracellular matrix (ECM) deposition, and macrophages integrate inflammatory signaling across the microenvironment.

A central finding is the suggested role of FOXP2+ chief cells in distinguishing fibrotic CBTs (FCBTs). These cells shift from hypoxia-responsive states in non-fibrotic tumors (nFCBTs) toward mitochondrial biogenesis and aerobic respiration in FCBTs, marked by FOXP2 upregulation. This metabolic reprogramming correlates with enhanced *MIF* expression, and we propose that this may activate the MIF-(CD74+CD44) axis to potentially recruit macrophages. Further experimental validation is required to confirm this putative mechanism. Such neuron-immune crosstalk establishes a pro-fibrotic feedback loop, positioning FOXP2 not merely as a biomarker but as a potential regulator of fibrosis initiation.

Concurrently, FCBTs exhibit significant expansion of endothelial cells, organized into functionally distinct subsets. Pseudotime analysis reveals a trajectory from adhesion-competent EC1 (SELE+/SELP+/ACKR1+) to angiogenic EC2/EC3 (KDR+/INSR+). Critically, macrophages amplify this process through CXCLs-ACKR1 signaling (dominant for EC1) and VEGFA-VEGFR/NAMPT-INSR pathways (active in EC2/EC3). This immune-vascular crosstalk fuels neovascularization—a hallmark of fibrotic microenvironments—and underscores macrophages as master coordinators of stromal remodeling.

The effector phase of fibrosis is driven by CXCL14+ fibroblasts, which are markedly enriched in FCBTs. CXCL14+ fibroblasts have been reported to promote ECM remodeling and collagen deposition in idiopathic pulmonary fibrosis, inflammatory bowel disease, and lung adenocarcinoma [27,28,29]. These cells exhibit pronounced upregulation of ECM-organization genes (e.g., OGN) and collagen biosynthesis pathways, directly linking to histologically confirmed collagen deposition. Our data extend these prior observations to CBTs, identifying CXCL14+ fibroblasts as a dominant matrix-producing subset in fibrotic CBTs. Lineage tracing indicates that CXCL14+ fibroblasts derive from SMC4, a smooth muscle subcluster expressing collagen-remodeling genes (ADAMTS2, AEBP1, FMOD). This transdifferentiation (SMC-fibroblast) parallels mechanisms in organ fibrosis, suggesting conserved pathways in CBT pathogenesis.

Beyond these core players, T/NK cells and mast cells further modulate the fibrotic niche. Though their proportions remain stable between FCBTs and nFCBTs, functional analyses reveal T/NK cell interactions with fibroblasts via TGFB1-(ACVR1+TGFBR1) and with ECs via CCL5-ACKR1, while mast cell subsets exhibit pro-angiogenic signatures. These interactions reinforce inflammation without altering cellular abundance, highlighting the importance of functional states over compositional changes.

Compared with prior studies that relied on bulk analysis or immunohistochemistry of CBTs [19,20,21], our single-cell resolution atlas reveals previously unappreciated cellular heterogeneity and dynamic inter-lineage transitions. While earlier work has implicated hypoxia and SDH mutations in paraganglioma pathogenesis [1,3,4], the specific role of FOXP2+ chief cells in driving a metabolic shift toward mitochondrial respiration and MIF-mediated macrophage recruitment has not been described. Furthermore, the transdifferentiation of smooth muscle cells into CXCL14+ matrix-producing fibroblasts, as suggested by our pseudotime analysis, represents a novel mechanism for ECM deposition in CBT fibrosis, distinct from classical fibroblast activation in other tumor types.

The multi-cellular communication network we delineated—from FOXP2+ chief cells (MIF) to macrophages (CXCLs) to endothelial cells (ACKR1/VEGFR) and finally to CXCL14+ fibroblasts (ECM production)—provides a stepwise model for fibrotic TME remodeling in CBTs. This axis links neuronal reprogramming, immune recruitment, pathological angiogenesis, and matrix deposition into a coherent pathogenic cascade. Disrupting any node of this network, for instance by targeting FOXP2 or CXCL14, could potentially halt or reverse fibrosis, thereby reducing surgical complications and improving clinical outcomes in CBT patients.

Our study has limitations. The sample size (n = 6) may not capture the full spectrum of CBT heterogeneity, and functional validation of FOXP2/CXCL14 roles requires in vivo models. Nevertheless, the identification of FOXP2+ chief cells as fibrosis initiators, SMC-derived fibroblasts as ECM effectors, and CXCL8/CXCL14 as immune-stromal signaling hubs offers actionable therapeutic targets. Finally, while our transcriptomic and histological data strongly implicate FOXP2+ chief cells and CXCL14+ fibroblasts in CBT fibrosis, causal inference (e.g., via MIF neutralization or CXCL14 perturbation) requires functional validation in future studies. Inhibiting these nodes could disrupt the vicious cycle of metabolic dysregulation, angiogenesis, and collagen deposition that characterizes advanced CBTs.

## 4. Materials and Methods

### 4.1. Human CBT Samples

This study was approved by the Ethics Committee of Peking University Third Hospital (IRB00006761-M2023748). Written informed consent was obtained from all participants. All experiments adhered to the WMA Declaration of Helsinki and the Belmont Report. Surgical biopsies were obtained from CBT patients (aged 20–45 years) diagnosed clinically and pathologically at the Peking University Third Hospital Neurosurgery Department.

### 4.2. Preparation of Single-Cell Suspensions

Fresh tissues were washed in ice-cold RPMI1640 and dissociated using Tissue Dissociation Reagent A (Seekone K01301-30, Beijing, China) according to the manufacturer’s instructions. DNase I (Sigma 9003-98-9, Darmstadt, Germany) was used if needed. After erythrocyte removal (Solarbio R1010, Beijing, China), cell count/viability was assessed using a fluorescence cell analyzer (Countstar^®^ Rigel S2, Shanghai, China) with AO/PI. Debris and dead cells were removed (Miltenyi 130-109-398/130-090-101, Cologne, Germany) as necessary. The cells were washed twice in RPMI1640 (Servicebio G4531, Wuhan, China) and resuspended in 1× PBS (Servicebio G4202, Wuhan, China) + 0.04% BSA (Servicebio GC305010, Wuhan, China) at 1 × 10^6^ cells/mL.

### 4.3. Single-Cell RNA-Seq Library Construction and Sequencing

Single-cell RNA-Seq libraries were prepared using Chromium Next GEM Single Cell 3′ Reagent Kits v3.1 (10× Genomics Catalog No. 100026). Briefly, an appropriate number of cells were mixed with reverse transcription reagent and then loaded into the sample well in Chromium Next GEM Chip G. Subsequently, Gel Beads and Partitioning Oil were dispensed into corresponding wells separately in the chip. After emulsion droplet generation, reverse transcription was performed at 53 °C for 45 min and inactivated at 85 °C for 5 min. Next, cDNA was purified from broken droplets and amplified in a PCR reaction. The amplified cDNA product was then cleaned, fragmented, end-repaired, A-tailed, and ligated to a sequencing adaptor. Finally, indexed PCR was performed to amplify the DNA representing the 3′ polyA part of expressing genes, which also contained a Cell Barcode and Unique Molecular Index. The indexed sequencing libraries were cleaned up with SPRI beads, quantified by quantitative PCR (KAPA Biosystems KK4824) and then sequenced on an Illumina NovaSeq 6000 with PE150 read length or a DNBSEQ-T7 platform with PE150 read length.

### 4.4. Processing of scRNA-Seq Data

Raw reads were aligned to the hg38 genome, and gene counts generated using Cell Ranger (v6.0.2). Seurat (v4.3.0) was used for QC: cells with <200 or >6000 genes detected or >20% mitochondrial reads were excluded. After QC, 64,944 cells were analyzed. Data normalization/scaling used SCTransform. Initial clustering (FindClusters, Louvain algorithm, resolution = 0.6, first 20 PCs) and UMAP visualization were performed. Cluster marker genes were identified (FindAllMarkers, Wilcoxon rank-sum test; |avg_logFC| ≥0.5, *p*_val_adj ≤ 0.05). For subclustering, major cell types were extracted and re-clustered (first 20 PCs, appropriate resolution). Subcluster markers were identified similarly. Heatmaps used DoHeatmap (top 20 markers).

### 4.5. RNA-Seq Library Construction and Sequencing

RNA library for RNA-seq was prepared as follows: mRNA was purified from total RNA using polyT and then fragmented into 300~350 bp fragments. The first strand cDNA was reverse-transcribed using fragmented RNA and dNTPs (dATP, dTTP, dCTP and dGTP) and second strand cDNA synthesis was subsequently performed. Remaining overhangs of double-strand cDNA were converted into blunt ends via exonuclease/polymerase activities. After adenylation of 3′ ends of DNA fragments, sequencing adaptors were ligated to the cDNA and the library fragments were purified. The template was enriched by PCR, and the PCR product was purified to obtain the final library. After library preparation and pooling of different samples, the samples were subjected to an NGS platform by Berry Genomics Co., Ltd. (http://www.berrygenomics.com/, Beijing, China).

### 4.6. Differential Gene Expression Analysis

Differentially expressed genes between nFCBT and FCBT were identified per cell type/subcluster using FindMarkers (min.pct > 0.25, thresh.use = 0.25; Wilcoxon rank-sum test; |avg_logFC| > 0.5, *p*_val_adj < 0.05). For differential expression, min.pct = 0.25 and thresh.use = 0.25 were used as pre-filters. Adjusted *p*-values were calculated with the Benjamini-Hochberg method. Gene expression plots were generated using Seurat’s FeaturePlot (Seurat v4.3.0), VlnPlot (Seurat v4.3.0), DoHeatmap (Seurat v4.3.0) and ggplot2 (v3.5.1). Gene expression plots were generated in R (v4.3.0).

### 4.7. Functional Enrichment Analysis

GO and KEGG enrichment analyses were performed using the clusterProfiler R package (v4.5.1.902). For GO analysis, the ‘C5’ collection from MSigDB was used; for KEGG, the ‘C2’ collection was used. *p*-Values were adjusted using the Benjamini-Hochberg method, and terms with an adjusted *p*-value < 0.05 were considered significantly enriched. Results were visualized with ggplot2 (v3.5.1).

### 4.8. Cell-Cell Communication Analysis

CellChat (v1.1.3) inferred communication networks (cell types > 10 cells). computeCommunProb used the ‘trimean’ method with 100 permutations; interactions with a permutation-based *p* < 0.05 were considered significant (no additional multiple-testing correction). Visualization functions included netVisual_aggregate, netVisual_heatmap, netVisual_bubble, and netVisual_circle.

### 4.9. Pseudotime Analysis

Trajectory analysis used Monocle3 post-QC (200–6000 genes/cell, <20% mt-genes) and SCTransform normalization. UMAP reduction (n_neighbors = 50) preceded graph construction with manual root cell annotation. Branch-associated genes were identified (using Moran’s I test). Pseudotime dynamics were modeled using GAMs. RNA velocity (scVelo) complemented trajectory inference, validated by bootstrapping and alternative methods (Slingshot, TSCAN).

### 4.10. Immunohistochemical (IHC) Staining

Deparaffinized sections were dehydrated, peroxidase-blocked (3% H_2_O_2_), and blocked (10% goat serum). Primary antibodies (FOXP2: Bioss bs-16173R polyclonal antibody 1:200; OGN: Solarbio K113016P polyclonal antibody 1:100; and CD31 Proteintech 66065-2-Ig Monoclonal antibody 1:5000) were applied overnight (4 °C). Sections were incubated with HRP-conjugated secondary antibodies (goat anti-rabbit IgG-HRP: Solarbio SE134 1:100; goat anti-mouse IgG-HRP: Solarbio SE131 1:100), visualized with DAB (Servicebio G1313), counterstained with hematoxylin, washed in PBS, and mounted. Imaging used a light microscope.

### 4.11. Immunofluorescence (IF) Staining

Deparaffinized, dehydrated, peroxidase-blocked sections were blocked (10% goat serum). Primary antibodies (FOXP2: Bioss bs-16173R polyclonal antibody 1:200; SYP: Proteintech 82900-1-RR recombinant antibody 1:1000; DCN: Proteintech 14667-1-AP polyclonal antibody 1:1000; CXCL14: Solarbio K114013P polyclonal antibody 1:100; OGN: Solarbio K113016P polyclonal antibody 1:100) were incubated overnight (4 °C). HRP-conjugated secondary antibodies (as above) were applied, followed by TSA staining (Servicebio G1236, room temperature, dark). Nuclei were counterstained with DAPI. Sections were washed in PBS and mounted with anti-fade medium. Imaging used fluorescence microscopy.

### 4.12. Masson Staining

Deparaffinized and dehydrated sections were stained using a Masson kit (Solarbio G1343) per the manufacturer’s instructions. Collagen fibers stained blue; muscle fibers stained red.

### 4.13. Statistical Analysis

A formal power calculation was not feasible due to the lack of prior scRNA-seq effect estimates in CBTs. The choice of n = 3 per group is consistent with published scRNA-seq studies aiming to discover cell-state heterogeneity in rare or surgically challenging tumors. Multiple testing was rigorously controlled using BH adjustment (FDR < 0.05) for all single-cell transcriptomic comparisons. Data were analyzed using IBM SPSS Statistics 23 and R (v4.3.0). Continuous variables are expressed as mean ± SEM. Normality was assessed via the Shapiro–Wilk test. For two-group comparisons (e.g., nFCBT vs. FCBT), unpaired two-tailed Student’s *t*-tests (normal data) or Mann–Whitney U tests (non-normal data) were applied; for multi-group comparisons, one-way ANOVA with Bonferroni post hoc tests or Kruskal–Wallis with Dunn’s tests were used. Single-cell analyses employed Seurat (v4.3.0) for differential expression (Wilcoxon rank-sum test; |avg_log2FC| > 0.5, Benjamini–Hochberg adjusted *p* < 0.05, min.pct > 0.25). Gene set enrichment (clusterProfiler v4.5.1.902) used two-sided permutation tests. Cell–cell communication (CellChat v1.1.3) considered interactions significant at *p* < 0.05 (trimean method). Pseudotime trajectories (Monocle3/scVelo) identified branch genes via Moran’s I test and modeled dynamics with GAMs. All tests were two-sided with significance at *p* < 0.05. Visualization used GraphPad Prism 8.0 and ggplot2 (v3.5.1).

## 5. Conclusions

In conclusion, this single-cell atlas deciphers how cellular crosstalk converges to drive CBT fibrosis. FOXP2-mediated neuronal reprogramming may recruit macrophages, which in turn ignite angiogenic EC expansion and SMC transdifferentiation into collagen-secreting fibroblasts. Targeting this axis—particularly FOXP2 (metabolic shift), CXCL14 (fibroblast activation), and CXCLs (macrophage-EC crosstalk)—may mitigate surgical complications and improve outcomes for patients with fibrotic CBTs.

## Data Availability

The datasets generated and/or analyzed during the current study are not publicly available due to patients’ privacy but are available from the corresponding author on reasonable request.

## Supplementary Materials

The following supporting information can be downloaded at https://www.mdpi.com/article/10.3390/ijms27135750/s1.
