# Supplementary material for: FOXP2+ Chief Cells and CXCL14+ Fibroblasts Drive Fibrotic Remodeling in Carotid Body Tumors
Source: Int J Mol Sci. 2026 Jun 25;27(13):5750. doi: 10.3390/ijms27135750 (PMC13361602; doi:10.3390/ijms27135750)
Supplement: Supplementary file 1 [file ijms-27-05750-s001.zip › ijms-4304803-supplementary.pdf]

Supporting Information for

FOXP2+ Chief Cells and CXCL14+ Fibroblasts Drive Fibrotic Remodeling in Carotid Body Tumors

Kangxi Cao 1; Jiazhi Yu 1; Guangnan Ao 1; Zongli Han 1; Zhongzheng Wang 1; Yunfeng Han 1; Tao Wang 1*

1 Department of Neurosurgery, Peking University Third Hospital, Beijing 100191, China

* corresponding email: wangtao@bjmu.edu.cn

Contents

1. Supporting Figures ………………………………………………………..…S2-S8

2. Supporting Table ……………………………………………………..……S9

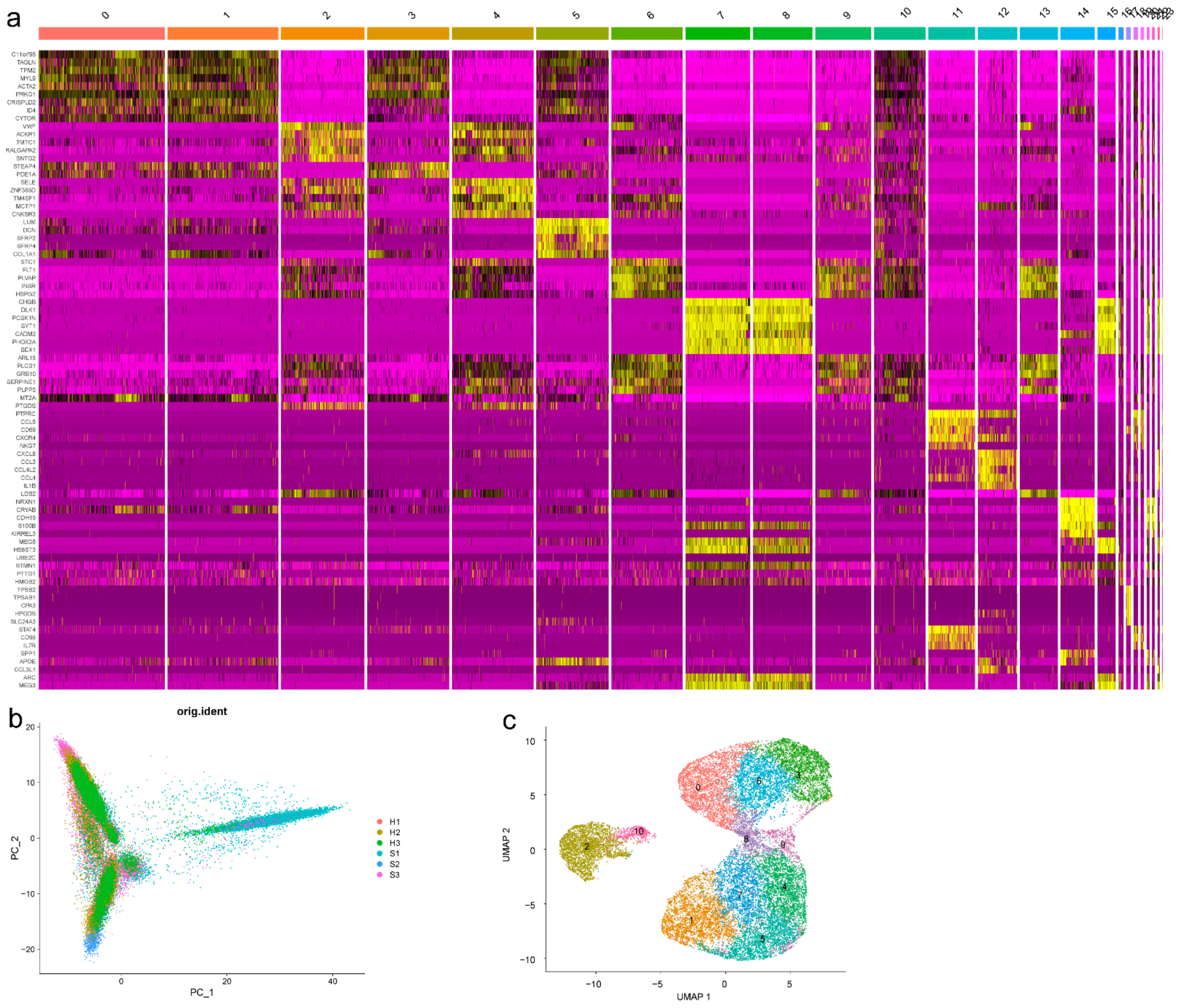
a
C11orf96
TAGLN
TPM2
MYL9
ACTA2
PRKG1
CRISPLD2
ID4
CYTOR
VWF
ACKR1
TMTC1
RALGAPA2
SNTG2
STEAP4
PDE1A
SELE
ZNF385D
TM4SF1
MCTP1
CNKSR3
LUM
DCN
SFRP2
SFRP4
COL1A1
STC1
FLT1
PLVAP
INSR
HSPG2
CHGB
DLK1
PCSK1N
SYT1
CADM2
PHOX2A
BEX1
ARL15
PLCB1
GRB10
SERPINE1
PLPP3
MT2A
PTGDS
PTPRC
CCL5
CD69
CXCR4
NKG7
CXCL8
CCL3
CCL4L2
CCL4
IL1B
LDB2
NRXN1
CRYAB
CDH19
S100B
KIRREL3
MEG8
HS6ST3
UBE2C
STMN1
PTTG1
HMGB2
TPSB2
TPSAB1
CPA3
HPGDS
SLC24A3
STAT4
CD96
IL7R
SPP1
APOE
CCL3L1
ARC
MEG3
b
orig.ident
PC_1
PC_2
H1
H2
H3
S1
S2
S3
c
UMAP 1
UMAP 2

S2

Supplementary Figure S1| scRNA-seq reveals cellular composition in CBT. (a) Heatmap showing the expression levels of marker genes in all clusters. (b) Cells are colored by samples and conditions' identity in the UMAP plots. (c) UMAP plot showing endothelia cells subclusters in nFCBT and FCBT.

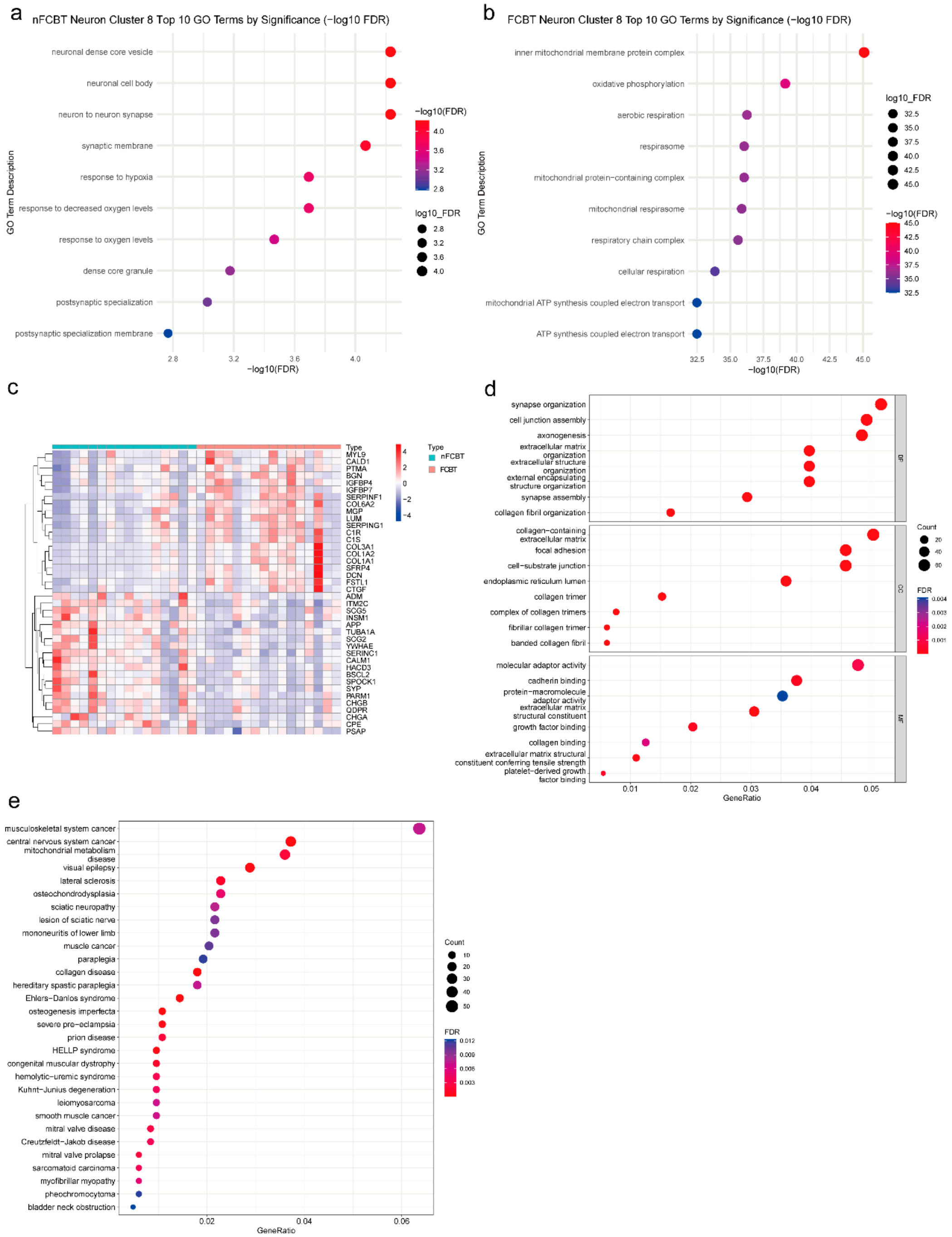

Supplementary Figure S2| Gene functional analysis of FOXP2. (a) Top enriched GO pathways of neurons cluster 8 in nFCBT (GSEA). (b) Top enriched GO pathways of neurons cluster 8 in FCBT (GSEA). (c) Heatmap of top 20 up and down-regulated

DEGs. (d) GO pathways of DEGs. (e) DO results of DEGs. NES: enrichment score; GSEA FDR was from Benjamini-Hochberg FDR correction.

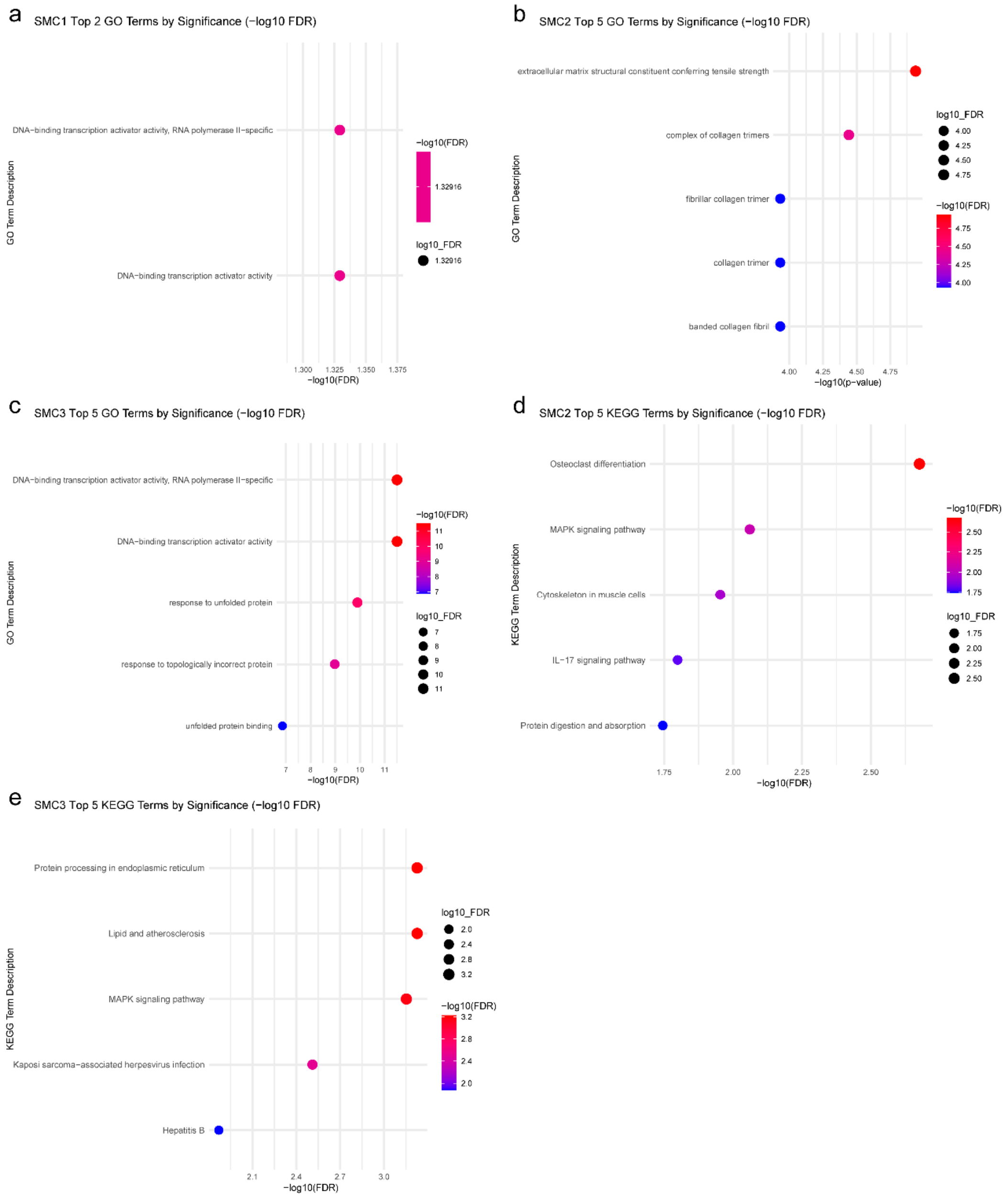

Supplementary Figure S3| GO and KEGG analyses of smooth muscle cells. (a) Top enriched GO pathways of SMC1 in nFCBT (GSEA). (b) Top enriched GO pathways of SMC2 in nFCBT (GSEA). (c) Top enriched GO pathways of SMC3 in nFCBT (GSEA). (d) Top enriched KEGG pathways of SMC2 in FCBT (GSEA). (e) Top enriched KEGG pathways of SMC3 in FCBT (GSEA). NES: enrichment score; GSEA FDR was from Benjamini-Hochberg FDR correction.

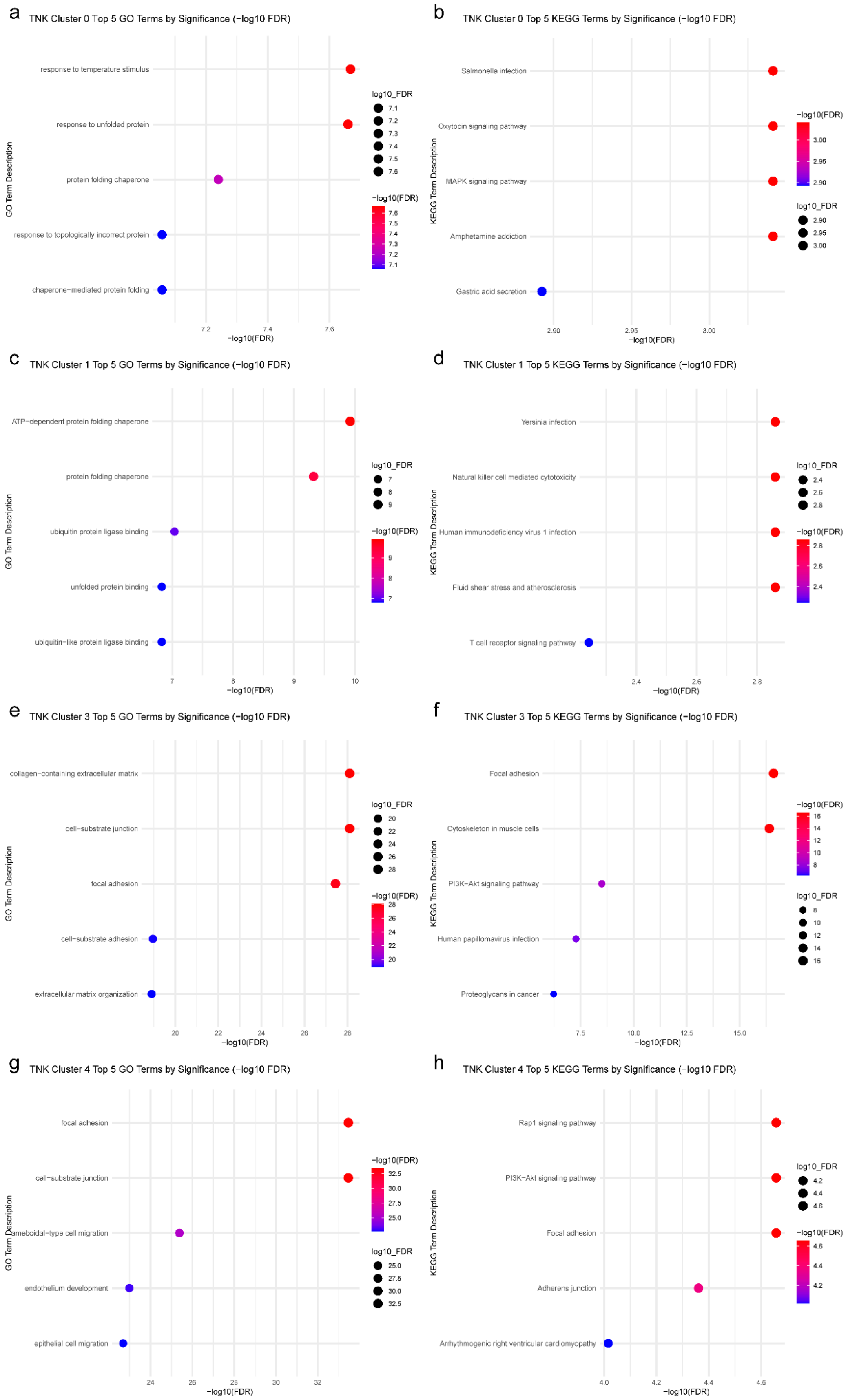
a
TNK Cluster 0 Top 5 GO Terms by Significance (−log10 FDR)
response to temperature stimulus
response to unfolded protein
protein folding chaperone
response to topologically incorrect protein
chaperone−mediated protein folding
GO Term Description
−log10(FDR)
log10_FDR
b
TNK Cluster 0 Top 5 KEGG Terms by Significance (−log10 FDR)
Salmonella infection
Oxytocin signaling pathway
MAPK signaling pathway
Amphetamine addiction
Gastric acid secretion
KEGG Term Description
−log10(FDR)
log10_FDR
c
TNK Cluster 1 Top 5 GO Terms by Significance (−log10 FDR)
ATP−dependent protein folding chaperone
protein folding chaperone
ubiquitin protein ligase binding
unfolded protein binding
ubiquitin−like protein ligase binding
GO Term Description
−log10(FDR)
log10_FDR
d
TNK Cluster 1 Top 5 KEGG Terms by Significance (−log10 FDR)
Yersinia infection
Natural killer cell mediated cytotoxicity
Human immunodeficiency virus 1 infection
Fluid shear stress and atherosclerosis
T cell receptor signaling pathway
KEGG Term Description
−log10(FDR)
log10_FDR
e
TNK Cluster 3 Top 5 GO Terms by Significance (−log10 FDR)
collagen−containing extracellular matrix
cell−substrate junction
focal adhesion
cell−substrate adhesion
extracellular matrix organization
GO Term Description
−log10(FDR)
log10_FDR
f
TNK Cluster 3 Top 5 KEGG Terms by Significance (−log10 FDR)
Focal adhesion
Cytoskeleton in muscle cells
PI3K−Akt signaling pathway
Human papillomavirus infection
Proteoglycans in cancer
KEGG Term Description
−log10(FDR)
log10_FDR
g
TNK Cluster 4 Top 5 GO Terms by Significance (−log10 FDR)
focal adhesion
cell−substrate junction
ameboidal−type cell migration
endothelium development
epithelial cell migration
GO Term Description
−log10(FDR)
log10_FDR
h
TNK Cluster 4 Top 5 KEGG Terms by Significance (−log10 FDR)
Rap1 signaling pathway
PI3K−Akt signaling pathway
Focal adhesion
Adherens junction
Arrhythmogenic right ventricular cardiomyopathy
KEGG Term Description
−log10(FDR)
log10_FDR

Supplementary Figure S4| GO and KEGG analyses of T and NK cells. (a) Top enriched GO pathways of TNK cluster 0 in nFCBT (GSEA). (b) Top enriched KEGG pathways of TNK cluster 0 in FCBT (GSEA). (c) Top enriched GO pathways of TNK cluster 1 in nFCBT (GSEA). (d) Top enriched KEGG pathways of TNK cluster 1 in FCBT (GSEA). (e) Top enriched GO pathways of TNK cluster 3 in nFCBT (GSEA). (f) Top enriched KEGG pathways of TNK cluster 3 in FCBT (GSEA). (g) Top enriched GO pathways of TNK cluster 4 in nFCBT (GSEA). (h) Top enriched KEGG pathways of TNK cluster 4 in FCBT (GSEA). NES: enrichment score; GSEA FDR was from Benjamini-Hochberg FDR correction.

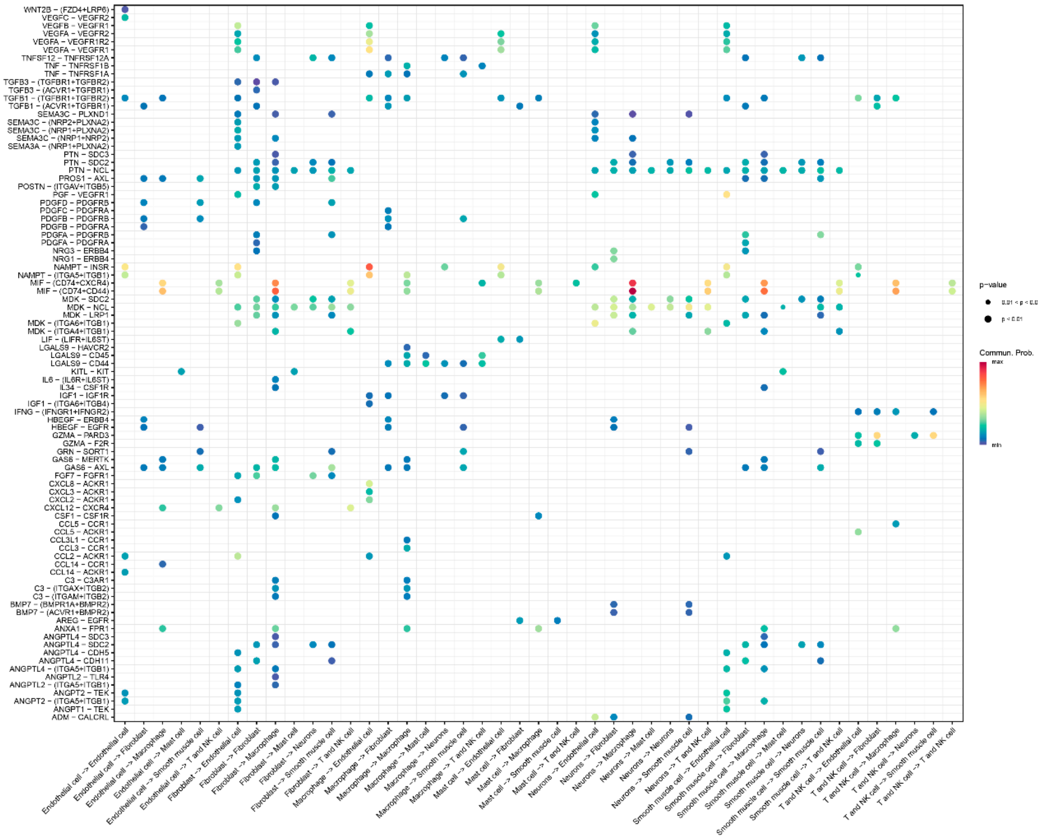

Supplementary Figure S5| Bubble plot: ligand-receptor interactions between all cell types.

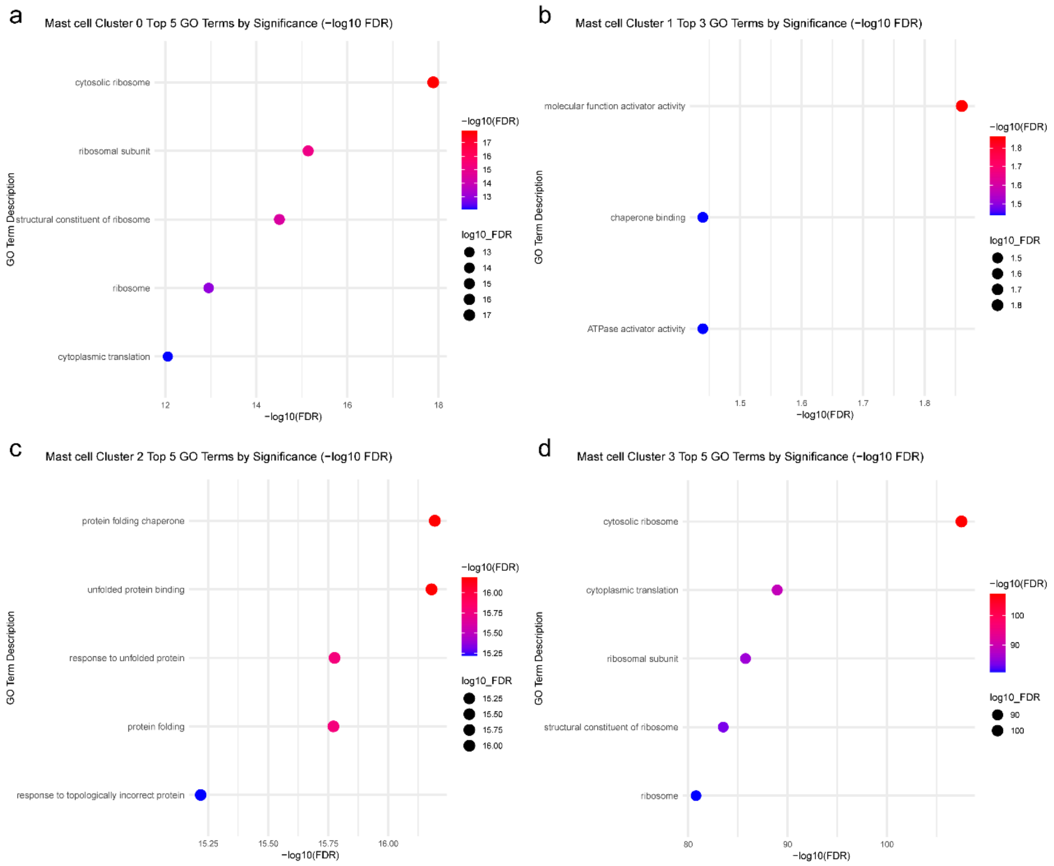

Supplementary Figure S6| GO analysis of mast cells. (a) Top enriched GO pathways of mast cluster 0 in CBT (GSEA). (b) Top enriched GO pathways of mast cluster 1 in CBT (GSEA). (c) Top enriched GO pathways of mast cluster 2 in CBT (GSEA). (d) Top enriched GO pathways of mast cluster 3 in CBT (GSEA). NES: enrichment score; GSEA FDR was from Benjamini-Hochberg FDR correction.

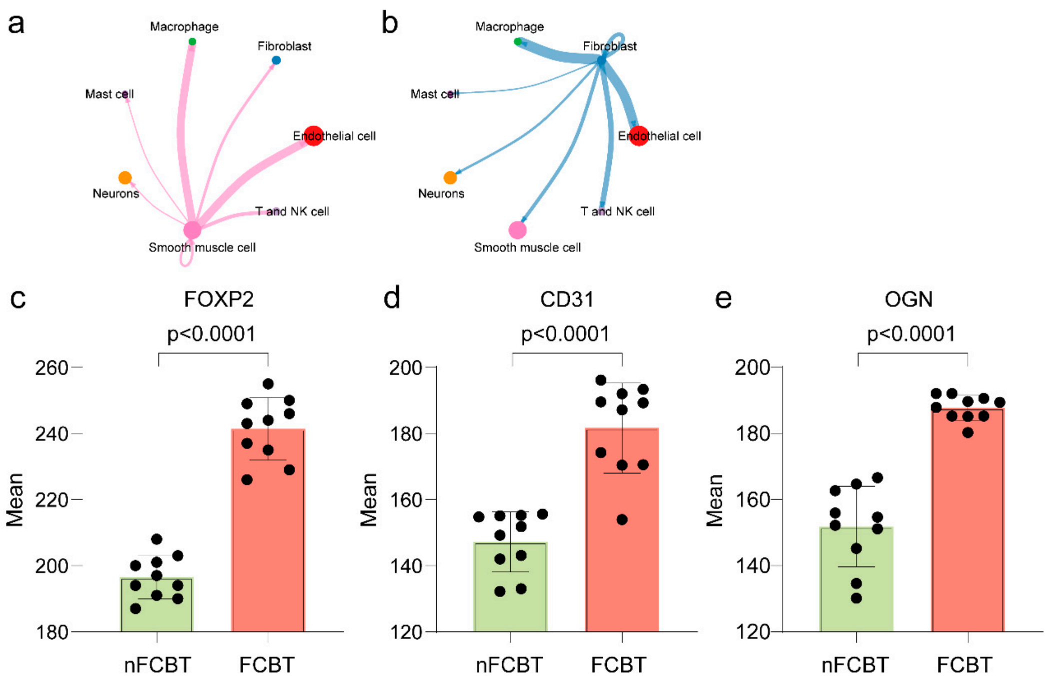

Supplementary Figure S7| (a, b) The cell chat results of smooth muscle cell and fibroblast, respectively. (c-e) The statistical analyses of FOXP2, CD31, and OGN, respectively. Data: mean ± SEM; p-value determined by two-tailed unpaired t-test (nFCBT vs FCBT) (c-e).

Supplementary Table S1 Top 5 markers of each fibroblast clusters.

p_val	avg_log2FC	pct.1	pct.2	p_val_adj	cluster	gene
4.04E-247	1.796405	0.864	0.443	1.48E-242	0	RGS5
2.10E-183	1.184682	0.733	0.324	7.69E-179	0	NFATC2
1.42E-175	1.291331	0.508	0.119	5.19E-171	0	NRG3
2.05E-162	1.426063	0.478	0.119	7.49E-158	0	LINC01060
1.89E-153	1.114167	0.818	0.529	6.92E-149	0	FNDC3A
0	4.141628	0.58	0.039	0	1	CXCL14
0	2.642092	0.723	0.091	0	1	C3
2.61E-296	3.660744	0.478	0.033	9.57E-292	1	PLA2G2A
1.12E-273	2.691278	0.578	0.096	4.10E-269	1	CFD
1.83E-191	2.55718	0.65	0.24	6.69E-187	1	APOD
1.56E-46	1.136044	0.94	0.928	5.72E-42	2	TENM4
3.02E-44	1.265207	0.601	0.417	1.10E-39	2	POSTN
1.02E-42	0.923307	0.537	0.39	3.74E-38	2	COL3A1
2.77E-28	0.939102	0.311	0.17	1.01E-23	2	RMRP
6.47E-23	1.130878	0.926	0.945	2.37E-18	2	COL1A1
3.87E-155	2.113678	0.647	0.122	1.42E-150	3	ADGRL3
5.77E-100	1.79769	0.742	0.261	2.11E-95	3	INPP4B
2.91E-98	2.70022	0.346	0.045	1.06E-93	3	CCL21
1.12E-93	2.829555	0.902	0.566	4.09E-89	3	ACTA2
5.38E-92	1.814452	0.974	0.807	1.97E-87	3	MYL9
